# Increased Responses of Phenoloxidase in Chlorantraniliprole Resistance of *Plutella xylostella* (Lepidoptera: Plutellidae)

**DOI:** 10.1093/jisesa/ieaa066

**Published:** 2020-07-03

**Authors:** Nian-Meng Wang, Jing-Jing Li, Ze-Yu Shang, Qi-Tong Yu, Chao-Bin Xue

**Affiliations:** Key Laboratory of Pesticide Toxicology and Application Technique, College of Plant Protection, Shandong Agricultural University, Tai’an, China

**Keywords:** phenoloxidase, immune defenses, chlorantraniliprole resistance, differential gene expression

## Abstract

The diamondback moth (*Plutella xylostella,* DBM) is an important pest of cruciferous vegetables. The use of chlorantraniliprole has been essential in the management of the DBM. However, in many countries and areas, DBM has become highly resistant to chlorantraniliprole. Three different DBM strains, susceptible (S), chlorantraniliprole-selected (R_c_), and field-collected (R_b_) resistant strains/populations were studied for the role of phenoloxidase in resistance development to the insecticide. By assaying the activity of phenoloxidase (PO) in the three different DBM strains, the results showed that the PO activity in the R_c_ strain was increased significantly compared with the S strain. The synergistic effects of quercetin showed that the resistant ratio (RR) of the QR_c_ larvae to chlorantraniliprole was decreased from 423.95 to 316.42-fold compared with the R_c_ larvae. Further studies demonstrated that the transcriptional and translational expression levels of *PxPPO1* (*P. xylostella* prophenoloxidase-1 gene) and *PxPPO2* (*P. xylostella* prophenoloxidase-2 gene) were increased to varying degrees compared with the S strain, such as the transcriptional expression levels of *PxPPO2* were 24.02-fold that of the S strain. The responses of phenoloxidase were significantly different in chlorantraniliprole-resistant DBM.

## Graphical Abstract

**Figure:**
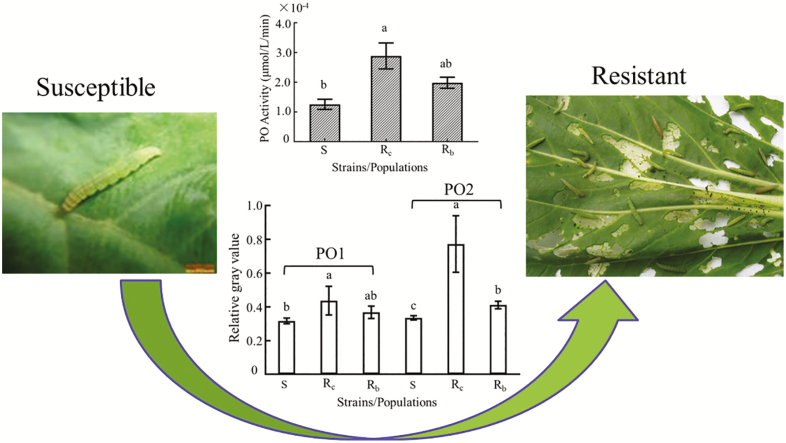

The diamondback moth (*Plutella xylostella*, DBM) (Lepidoptera: Plutellidae) has high fecundity and a rapid development time. It can produce multiple generations in a year and cause severe damage to cruciferous vegetables. The yearly worldwide cost of preventing and treating DBM infestations and their direct economic losses is approximately $ 4–5 billion US dollars (Zalucki et al. 2012, Sun et al. 2015). DBM populations have developed resistance to more than 93 types of insecticides (Whalon et al. 2019) and this presents significant challenges for their control.

Chlorantraniliprole and other diamide insecticides have unique mechanisms and demonstrate good control of Lepidoptera pests (Zhang et al. 2014; Xu et al. 2016, 2017; He et al. 2019), such as in DBM management (Nauen and Steinbach 2016). However, the resistance of DBM to this class of insecticides has become increasingly serious (Steinbach et al. 2015; Liu et al. 2015a,b; Mallott et al. 2019). This poses a challenge for the integrated management of DBM on vegetables. Currently, the target-site mutation (G4946E) in the trans-membrane domain of the ryanodine receptors (RyR) is considered a mainstream resistance mechanism in DBM, causing a serious resistance to chlorantraniliprole in the field populations of *P. xylostella* (Troczka et al. 2012, 2017;Gong et al. 2014). In addition, other mutations (resulted in E1338D, Q4594L, and I4790M [I4790K]) may coordinate mutations in RyR (Guo et al. 2014, Jourakua et al. 2020). Besides, the mRNA expression changes of *PxRyR* and cytochrome P450 *CYP6BG1* (Yan et al. 2014, Qin et al. 2018, Li et al. 2018) are also important resistant mechanisms of *P. xylostella* to chlorantraniliprole.

Phenoloxidase (PO) (EC1.14.18.1), also known as tyrosine hydroxylase or tyrosinase, is a member of the type-3-copper-containing proteins (Aguilera et al. 2013). In insects, PO is an important immune system protein. It usually exists as an inactive pro-phenoloxidase (PPO) that is stored in the hemolymph, midgut, and epidermal tissues (Lu et al. 2014). When insects are invaded by foreign objects such as parasites, humoral and cellular immunity are the main means of defense and PO plays an important role in this process (Shao et al. 2012). Insects, such as *Ostrinia furnacalis*, *Spodoptera exigua*, and *Bombyx mori* have developed resistance to insecticides, PO activity in the hemolymph was increased (Wu and Shang 1992, Wang 2005, Tang et al. 2016). Studies found that the activities of phenoloxidase were significantly increased in Cry1Ac-resistance strains of *P. xylostella*, and the higher PO activity may be attributed to the stress of Cry1Ac toxin (Liu et al. 2019). Thus, we want to know what the responses of phenoloxidase and its related genes, and that of the response whether related to the chemical insecticides resistance. So we used susceptible (S), lab-selected chlorantraniliprole (R_c_), and field-collected (R_b_) resistant strains/populations of DBM as the test insects, analyzed the phenoloxidase activity, transcriptional and translational expression levels of *PxPPO1* (*P. xylostella* prophenoloxidase-1 gene) and *PxPPO2* (*P. xylostella* prophenoloxidase-2 gene) in these resistant insects.

## Materials and Methods

### Insects

The susceptible strains (S) of DBM were collected in 2006 from the vegetable fields in south campus of the Shandong Agricultural University. The moths were continuously reared on cabbage seedlings in a laboratory setting with no insecticide contact. The chlorantraniliprole-selected (R_c_) resistant strain of DBM was obtained from partial S strain by lab-selection through multiple generations using chlorantraniliprole with LC_25_ concentration. The field-resistant population (R_b_) with 48-fold resistance was collected from Guangzhou Baiyun area, reared indoors without any insecticides (Table 1).

**Table 1.** The resistance ratio and ryanodine receptor allele frequencies in different strains/populations of *P. xylostella*^*a*^

Strain/ population	Characteristic	LD_50_ (µg/g) (95% FL)	RR^*b*^	RyR allele frequencies		
				G4946	G4946E	E4946V
S	Non-selected	0.87 (0.24~1.48)	—	100%	0	0
R_c_	Chlorantraniliprole-selected	368.65 (239.25 ~ 875.24)	423.95	100%	0	0
R_b_	Field-collected from Baiyun area of Guangdong, China	55.20 (49.52 ~61.94)	48.00	0	70%	30%

^*a*^Part of data taken from our previous studies of Qin et al. 2018.

^*b*^RR(Resistance Ratio) = LD_50_ of resistant strain/LD_50_ of susceptible strain.

### Chemicals

HRP-conjugated goat anti-rabbit antibodies (secondary antibody) and internal reference antibody (β-actin) were purchased from Vazyme Biotech Co. Ltd (Nanjing, Jiangsu, China). ECL substrate, SageBrightness West Pico Plus Chemiluminescence Substrate, was purchased from Sage Creation Science Co. Ltd (Beijing, China). L-DOPA and quercetin were purchased from Sigma–Aldrich (St. Louis, MO). Chlorantraniliprole (95.0%) was provided by the Institute for the Control of Agrochemicals, the Ministry of Agriculture (ICAMA), China. All other reagents were analytically pure.

### Phenoloxidase Extraction from DBM and Quantitation of Activity

The phenoloxidase extraction and enzyme activity determination were according to our previous studies (Xue et al. 2007). The third-instar larvae of DBM were homogenized in 0.02 mol/liter phosphate buffer (pH 6.5) at 5 mg larvae/ml. The homogenates were transferred to precooled centrifuge tubes and centrifuged at 4°C and 9,310 × *g* for 30 min. The supernatant was collected and 40% ammonium sulfate was added before allowing it to stand for 30 min at 4°C. The solution was then centrifuged for 30 min at 9,310 × *g* and 4°C. The precipitate was collected and dissolved in phosphate buffer prior to transfer to a dialysis bag for dialysis. During dialysis, the dialysate was changed multiple times until no sulfate ions (SO_4_^2−^) were detected. The enzyme solution was collected and stored at 4°C.

The 200 µl enzyme reaction solution contained 150 µl of phosphate buffer with a final concentration of 0.02 mol/liter and 40 µl of L-DOPA with a final concentration of 10 mmol/liter. The system was incubated at 37°C for 30 min and 10 µl enzyme solution was then added. A spectrophotometer (Epoch 2, Biotek Laboratories Inc., USA) was used to measure changes in absorbance with time at a wavelength of 490 nm (extinction coefficient, ε = 3,700 mol/liter·cm) (Jiménez et al. 2001). Absorbance was read every 30 s and the test period was 4 min. Enzyme activity was obtained from the gradient of the straight line. The enzyme assays was replicated three times in independent biological experiments.

### Synergism of Quercetin

Quercetin is a naturally occurring flavonoid, and can be obtained from quercus bark and sophora flower. It has significant inhibitory effects on phenoloxidase (Chen and Kubo 2002, Wang et al. 2005). Twenty larvae of the generations R_c57_, R_c58_, R_c59_, and R_c60_ strain were collected, respectively, and they fed on cabbage leaves that had been dipped in 10 mg/ml quercetin for 24 h separately. The treated larvae named QR_c57_, QR_c58_, QR_c59_, and QR_c60_, respectively. And the toxicity of chlorantraniliprole in every generation of QR_c_ and R_c_ strains with 20 larvae was assayed by the topical application according to the previous studies, respectively (Qin et al. 2018). The test was replicated three times.

### Real-time Fluorescence Quantitative PCR (qPCR)

Trizol was used to extract the total RNA from the hemolymph, which obtained by squeezing the third-instar DBM larvae. Twenty larvae were used for the qPCR for each strain. Reverse transcription was carried out with 2 μg of RNA samples using the FastQuant RT Kit (with gDNase) (Tiangen Biotech Co. Ltd, Beijing, China), following manufacturer instructions. The synthesized cDNA template was used for qPCR. The final qPCR reaction (20 µl) included 2 µl cDNA, 10 µl 2×SuperReal PreMix Plus (SYBR Green) (Tiangen Biotech Co. Ltd, Beijing, China), 0.6 µl each of forward and reverse primers (Table 2), and 6.8 µl ddH_2_O. The mean of the C_t_-values of the internal reference genes *RPS-13* (AY174891) and β*-actin* (AB282645) was used as a standard to compare the relative expression of target genes in the different strains. The qPCR conditions were: predenaturation at 95°C for 15 min; followed by 40 cycles of denaturation at 95°C for 15 s, annealing at 55°C for 20 s, and extension at 72°C for 30 s. The fluorescence signal was collected at 60°C and a melting curve was also obtained for verifying amplification specificity. The 2^-△△Ct^ method (Schmittgen and Livak 2008) was used to calculate the relative expression of *PxPPO1* (GU199189) and *PxPPO2* (ACS36209) in the different DBM strains. The expression levels of the target genes in the S strain were set as 1.0, and that of the resistant strain were their ratios. The qPCR for each sample was conducted with three replicates.

**Table 2.** List of primers and their sequences (Qin et al. 2018)

Gene	Primer sequences (5’–3’)	
	Forward primer	Reverse primer
*PxPPO1*	CGTCCATCATCAGCCGCAACC	TCTCCCATCACGCCGAATT
*PxPPO2*	AGCAGATGGCTGACGAGG	CGCAAAGTTGGGAATGG
β*-actin*	GGAGTGATGGTCGGTATGGGA	CGTTGTAGAAGGTGTGGTGCC
*RPS13*	TCAGGCTTATTCTCGTCG	GCTGTGCTGGATTCGTAC

### Western Blotting

Trizol was used to extract the total protein from the third-instar larvae. SDS–PAGE gels consisted of a 5% stacking gel and a 10% resolving gel. The 20 μg protein samples were loaded onto a PAGE gel. After sample loading, electrophoresis was conducted at constant voltage of 80 V for 1 h and the voltage was adjusted to 120 V after the bromophenol blue dye entered the resolving gel. Electrophoresis was carried out for 2–3 h until the bromophenol blue dye was 1.5 cm from the bottom of the gel. Proteins on the gel were transferred into PVDF membranes (0.45 μm Merck Millipore, Shanghai, China) and the transfer condition was 69 V for 2 h. After membrane transfer was completed, the membrane at the 50–60 kDa was cut out and immersed in a blocking solution (5% skim milk in TBST buffer) at room temperature for 2 h. Subsequently, target primary antibodies and internal reference primary antibodies were added according to the size of the proteins on the membrane (target primary antibody 1∶5000, internal reference antibody (β-actin 1∶2000) and incubated overnight at 4°C with shaking (Nantong, Jiangsu, China). The membranes were then washed for 15 min with TBST and this was repeated three times. The membrane was placed in a secondary antibody solution and incubated at room temperature for 2 h. The membranes were washed 15 min with TBST and this was repeated three times, and visualized by enhanced chemiluminescence using the ECL substrate. A freshly prepared luminescence working solution was used to soak the PVDF membrane. Following that, the membrane was placed in an exposure cassette and the air bubbles and creases were removed before exposure. A gel image processing system (ChampChemi Top420, Beijing, China) was used to analyze the molecular weight and grayscale values of target bands. Each sample was replicated three times.

### Data Analysis

We statistically analyzed data using analysis of variance (ANOVA) and evaluated differences in the means by Tukey’s multiple comparison test (*P* < 0.05) by using SPSS 16.0 (SPSS Inc., Chicago, IL). The different lowercase letters in the figures represent a significant difference (*P* < 0.05) between the different strains/populations.

## Results

### PO Activity in Larvae of Resistant DBMs

**Fig. 1.**
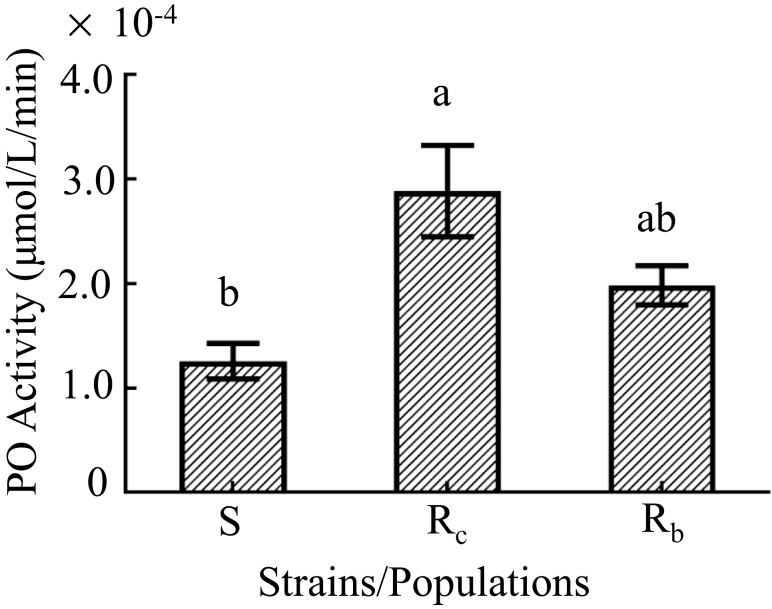
Phenoloxidase activities in different strains/populations of *P. xylostella*. The S, R_c_, and R_b_ are representative of the susceptible strain, chlorantraniliprole-selected resistant strain, and Baiyun field-collected resistant population, respectively. The error bars in the figure indicate SE.

We tested the PO activity of the three DBM strains larvae. The R_c_ DBM strain, produced by laboratory selection, had the highest PO activity, which was 2.295 times higher than that of the S strain. The R_c_ had 423.95-fold resistance than the S strain. The field-collected R_b_ resistant population was reared in laboratory culture without any insecticides. The PO activity was 1.577 times higher than that of the S strain (Fig. 1).

### Quercetin Synergism on Chlorantraniliprole Susceptibility

In order to find out the direct relationship between PO and resistance, the synergism experiments had been performed. Toxicity of chlorantraniliprole to the R_c_ strain larvae, pretreated with quercetin, was assayed, and the resistant ratio (RR) results showed that the RR of *P. xylostella* to chlorantraniliprole decreased from 423.95- to 316.42-fold, compared with the same generation of control larvae (Table 3).

**Table 3.** Synergistic effects of quercetin in the chlorantraniliprole-selected resistant *P. xylostella* strains

Generation	LD_50_ (µg/g)	95% FL (µg/g)	Slope (±SE)	χ ^2^	*P*	RR	Decreased RR
R_c57_	368.65	239.25–875.24	1.266 ± 0.314	0.93	0.95	423.95	—
R_c58_	387.31	256.34–984.57	1.421 ± 0.328	0.68	0.97	445.40	—
R_c59_	399.36	267.62–948.36	1.543 ± 0.341	2.25	0.90	459.26	—
R_c60_	411.35	264.74–1168.99	1.371 ± 0.328	0.94	0.96	473.06	—
QR_c57_^*a*^	275.15	185.76–725.58	1.398 ± 0.381	1.56	0.91	316.42	107.53^*b*^
QR_c58_	302.32	218.98–557.49	1.593 ± 0.325	0.64	0.98	347.37	98.03
QR_c59_	326.70	222.05–776.56	1.332 ± 0.313	1.65	0.93	376.36	82.90
QR_c60_	331.61	231.67–693.78	1.495 ± 0.325	0.74	0.97	381.02	92.04

^*a*^Generation from QR_c57_ to QR_c60_ is the resistance development of the R_c_ strains were feeding on cabbage leaves that had been dipped in 10 mg/ml quercetin for 24 h separately.

^*b*^Decreased resistance ratio, RR = R_c57_–QR_c57_.

### *PxPPO* mRNA Transcriptional Expression in Resistant DBM

**Fig. 2.**
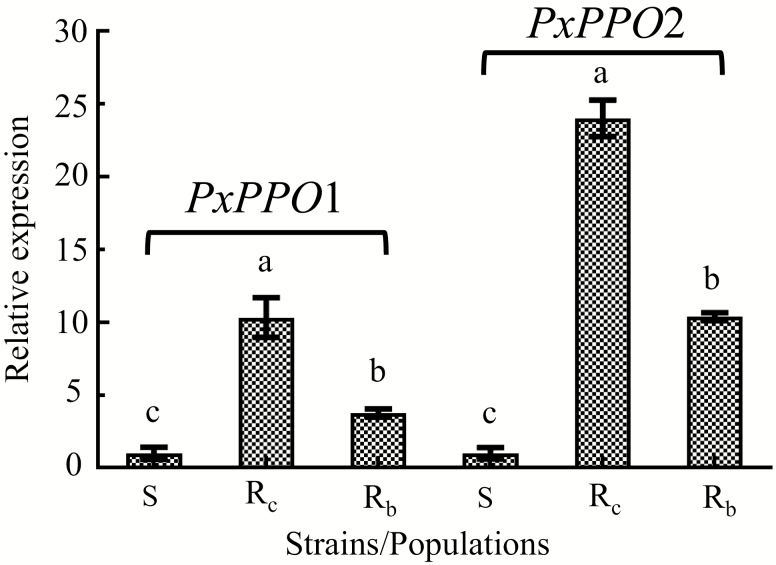
*PxPPO* relative expression levels in different strains/populations of *P. xylostella*. The S, R_c_, and R_b_ are representative of the susceptible strain, chlorantraniliprole-selected resistant strain, and Baiyun field-collected resistant population, respectively. The error bars in the figure indicated SE.

Real-time fluorescence quantitative PCR was used to validate the expression of *PxPPO1* and *PxPPO2* genes in the three DBM strains. The results showed that *PxPPO1* and *PxPPO2* expression was higher in the R_c_ strain, and it was 10.327 and 24.019 times higher than that of the S strain, respectively. *PxPPO1* and *PxPPO2* expression in the R_b_ populations was 3.764 and 10.402 times higher than that of the S strain, respectively (Fig. 2). The relative expressions of the *PxPPO1* and *PxPPO2* genes in the resistant strains/populations were significantly higher than the S strain.

### PO Protein Concentration in Resistant DBM

**Fig. 3.**
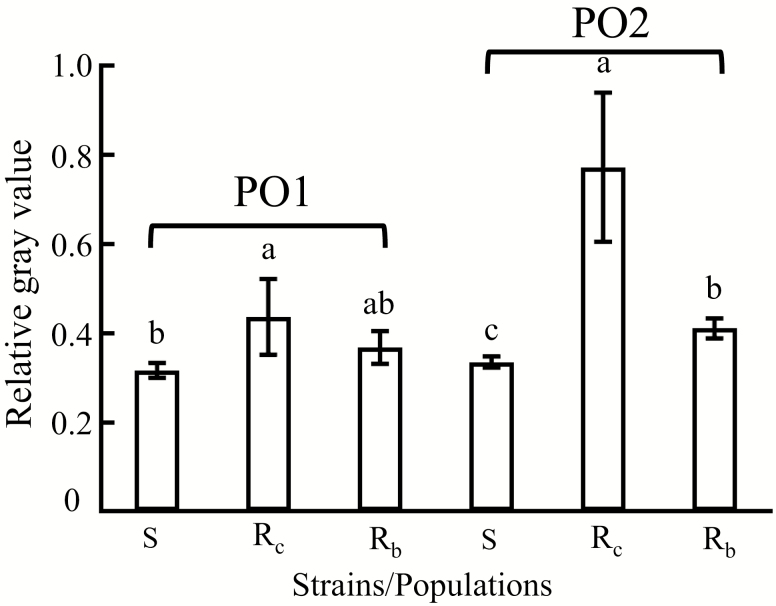
PO relative expression levels in different strains/populations of *P. xylostella. *The S, R_c_, and R_b_ are representative of the susceptible strain, chlorantraniliprole-selected resistant strain, and Baiyun field-collected resistant population, respectively. The error bars in the figure indicate SE.

Western blotting was used to quantify PO1 and PO2 protein levels in the tested DBM strains. From calculation of the relative grayscale value of electrophoretic bands, we found that PO1 and PO2 protein levels in the R_c_ strain were 1.378 and 2.303 times that of the S strain, respectively. And the PO1 and PO2 protein levels in the R_b_ strain were 1.159 and 1.225 times that of the S strain, respectively (Fig. 3). PO protein levels in the tested resistant strains were all significantly higher than the S strain.

## Discussion

Phenoloxidase plays an important role in the growth and development of insects and is a key enzyme for melanin synthesis. Phenoloxidase also plays a role in insect immune processes and PO activity is regarded as an important marker of host immunity (Cerenius and Söderhäll 2004, Nappi and Christensen 2005). After *B. mori* larvae were infected with the silkworm nuclear polyhedrosis virus (BmNPV), their hemolymph PO activity was significantly higher than the control group (Tang et al. 2016). When chlorfluazuron and diflubenzuron were added to artificial diet and fed to larvae of the *O. furnacalis*, their epidermal PO activity was significantly increased (Wu and Shang 1992). After treatment of *Musca domestica* larvae with chlorbenzuron, chitinase and phenoloxidase activities were both elevated (Ishaaya and Casida 1974). Insects regulate immune defense factors (including PO) to resist pathogen invasion and this is an important defense mechanism. When 0.14 mg/kg tebufenozide was used to treat the third-instar larvae of *S. exigua* for 24, 48, and 72 h, the epidermal PO enzyme activity first decreased, then increased, and finally decreased. This may be because after the insecticide has entered the insect, the toxic effects initially cause PO activity to decrease. However, the insect regulates its immune response, using elevated phenoloxidase activity, to gradually resist the effects of the toxin, until equilibrium was reached (Wang 2005). This study quantified PO activity in two different resistant DBM strains and demonstrated that they all had higher PO activity than the susceptible strain. Phenoloxidase has significant responses in chlorantraniliprole-resistance of *P. xylostella*.

*PxPPO1* and *PxPPO2* were upregulated in resistant DBM strains compared with a susceptible strain. This situation was also reported in the fourth-instar larvae of *Pieris rapae* infected with *Beauveria bassiana*. *PrPPO1* expression levels were significantly decreased at an infection time of 6–12 h, were similar to the control group at 24–48 h, and greatly increased after 72 h of infection (Lu et al. 2015). This demonstrates a ‘seesaw effect’ between insecticide toxicity and the immune defense process of the insect. After *B. mori* larvae were infected with BmNPV for 6–9 h, PPO1 and PPO2 genes both showed significant upregulation, while expression levels were significantly decreased at 24 h (Tang et al. 2016). In deltamethrin-resistant *Culex pipiens pallens*, the PO expression level in the resistant strains was significantly higher than susceptible strains (Cheng et al. 2011). The PO gene expression levels in deltamethrin-resistant and field-resistant houseflies were 13.38 and 6.24 times the levels of susceptible strains. In addition, PO gene expression levels and the LC_50_ of insecticides in field resistant housefly populations showed a linear correlation (Liu and Zhang 2011). In chlorantraniliprole-resistant DBM, the expression levels of four phenoloxidase unigenes were significantly upregulated with increasing resistance levels, and this is an important factor in the generation of chlorantraniliprole resistance in DBM (Lin et al. 2013). In chlorpyrifos- and fipronil-resistant DBM, the expression of phenoloxidase genes was also significantly upregulated compared with susceptible strains (Xia et al. 2015). This situation was also reported in drug resistance of *Culex pipiens* mosquitoes (Vézilier et al. 2013). After *Plasmodium yoelii* infects *Anopheles stephensi* mosquitoes, western blotting showed that the PO protein band in the midgut was significantly enhanced and midgut PO levels were significantly increased (Qin et al. 2004). After 24 h of *P. yoelii* infection in *Anopheles dirus*, the hemolymph PO protein levels were increased (Yang et al. 2004).

The results of this study show that PO activity, and mRNA transcriptional expression and protein levels of the PPO gene in resistant strains were all significantly higher than the susceptible strain. The responses of phenoloxidase may be the natural response of resistant DBM or an important factor in the formation of chlorantraniliprole-resistance, which need to be studied further.
